# Current treatment strategies for primary upper extremity deep venous thrombosis; a retrospective observational multicenter case series

**DOI:** 10.3389/fsurg.2022.1080584

**Published:** 2022-12-22

**Authors:** R. J. C. M. F. de Kleijn, L. Schropp, J. Westerink, M. Nijkeuter, J. van Laanen, J. Teijink, C. Ünlu, A. W. F. Vos, E. S. van Hattum, B. J. Petri, G. J. de Borst

**Affiliations:** ^1^ Department of Vascular Surgery, University Medical Center Utrecht, Utrecht Netherlands; ^2^ Department of Internal Medicine, Isala Clinic, Zwolle, Netherlands; ^3^ Department of Vascular Medicine, University Medical Center Utrecht, Utrecht Netherlands; ^4^ Department of Vascular Surgery, Maastricht University Medical Center, Maastricht Netherlands; ^5^ Department of Vascular Surgery, Catharina Hospital, Eindhoven, Netherlands; ^6^ Department of Vascular Surgery, Noordwest-Ziekenhuisgroep, Alkmaar, Netherlands; ^7^ Department of Vascular Surgery, Antonius Hospital, Nieuwegein, Netherlands

**Keywords:** upper extremity deep venous thrombosis, UEDVT, venous thoracic outlet syndrome, first rib resection and scalenectomy, anticoagulation therapy

## Abstract

**Introduction:**

Current treatment strategies for primary upper extremity deep venous thrombosis (pUEDVT) range from conservative treatment with anticoagulation therapy to invasive treatment with thoracic outlet decompression surgery (TOD), frequently combined with catheter directed thrombolysis, percutaneous transluminal angioplasty, or stenting. Due to a lack of large prospective series with uniform data collection or a randomized trial, the optimal treatment strategy is still under debate. We conducted a multicenter observational study to assess the efficacy and safety of both the conservative and invasive treatment strategies for patients with pUEDVT.

**Methods:**

We retrospectively collected data from patients treated in five vascular referral and teaching hospitals in the Netherlands between 2008 and 2019. Patients were divided into a conservative (Group 1), an invasive treatment group (Group 2) and a cross-over group (Group 3) of patients who received surgical treatment after initial conservative therapy. Follow-up consisted of outpatient clinic visits and an electronic survey. Primary outcome was symptom free survival defined as absence of any symptom of the affected arm reported at last follow-up regardless of severity, or extent of functional disability. Secondary outcomes were incidence of bleeding complications, recurrent venous thromboembolism, surgical complications, and reinterventions.

**Results:**

A total of 115 patients were included (group 1 (*N* = 45), group 2 (*N* = 53) or group 3 (*N* = 27). The symptom free survival was 35.6%, 54.7% and 48.1% after a median follow-up of 36, 26 and 22 months in groups 1, 2 and 3 respectively. Incidence of bleeding complications was 8.6%, 3.8% and 18.5% and recurrent thrombosis occurred in 15.6%, 13.2% and 14.8% in groups 1–3 respectively.

**Conclusion:**

In this multicenter retrospective observational cohort analysis the conservative and direct invasive treatments for pUEDVT were deemed safe with low percentages of bleeding complications. Symptom free survival was highest in the direct surgical treatment group but still modest in all subgroups. Perioperative complications were infrequent with no related long term morbidity. Of relevance, pUEDVT patients with confirmed VTOS and recurrent symptoms after conservative treatment may still benefit from TOD surgery. However, symptom free survival of this delayed TOD seems lower than direct surgical treatment and bleeding complications seem to occur more frequently.

## Introduction

Upper extremity deep venous thrombosis can be divided in primary upper extremity deep venous thrombosis (pUEDVT) and secondary upper extremity deep venous thrombosis (sUEDVT). In sUEDVT a clear cause for thrombosis can be found; mostly indwelling devices such as intravenous catheters and pacemaker leads. In pUEDVT this clear cause for thrombosis is lacking. Primary upper extremity deep venous thrombosis (pUEDVT) can be divided into two subcategories; venous thoracic outlet syndrome (VTOS) (including the effort induced Paget-Schroetter syndrome) and idiopathic thrombosis. The incidence of pUEDVT is unclear, and estimates range between 0.5–3/100.000 people per year (1–3).

Currently implemented treatment strategies for pUEDVT can be roughly divided into a conservative and invasive approach. The conservative treatment consists of anticoagulation therapy, preferably Direct Oral Anticoagulants (DOAC) for at least 3–6 months but often for a prolonged period of time, combined with elastic stockings. Invasive treatment consists of thoracic outlet decompression surgery (TOD), and in the presence of acute thrombosis it can be preceded by catheter directed thrombolysis (CDT). Additional percutaneous transluminal angioplasty (PTA) and/or venous stenting might be used in selected cases (4).

Due to a lack of randomized controlled trials or prospective series with long-term follow-up, it remains unclear which approach is superior. Moreover, there is no consensus on a suitable primary outcome measure to compare the effect of both treatments. The most frequently used primary endpoint is post thrombotic syndrome (PTS) free survival because it is known for its chronic nature and long lasting reduction in quality of life (QoL) (5, 6). So far there is no validated scoring method to diagnose the presence or severity of PTS in the upper extremity. Thus, a wide array of definitions and scoring methods for PTS are used, making it difficult to compare study results. Based on small, monocentric, and retrospective case series the PTS incidence is estimated to be higher in the conservative treatment group (24%–66%) compared to the invasive treatment group (5%–24%) (7–12). A downside of the invasive strategy seems to be the higher risk of bleeding complications in addition to other surgery related complications that can occur in up to 25% of patients (7–9, 13–15). We conducted a multicenter retrospective observational cohort study to assess the efficacy and safety of the currently used diagnosis- and treatment strategies for patients with pUEDVT.

## Methods

Between 2008 and 2019, by using diagnosis treatment codes (DTC), we retrospectively identified all patients who were either diagnosed with pUEDVT or VTOS in five vascular referral and teaching hospitals in the Netherlands. Diagnosis of UEDVT was based on signs and symptoms combined with at least one imaging modality (Duplex, CT, MRI or conventional venography). Additionally for the diagnosis VTOS we adhered to the latest reporting standards for thoracic outlet syndrome (16); a significant compression of the subclavian vein with or without underlying damaging of the vein had to be present on contrast enhanced positional CT or MRI or venography. We identified all pUEDVT patients by reviewing electronic patient files and radiological exams and reports, making sure to exclude all patients with sUEDVT (malignancies, indwelling vascular devices etc.). Follow-up data was collected through electronic patient files and an online survey at last follow-up informing about residual symptoms, anticoagulant usage, hospital admission, reinterventions and adverse events. Patient follow-up was recorded up until 2021. Follow-up was defined as the time between start of treatment until the last reported hospital visit or date of completion of the online survey.

The cohort was divided into a conservative and an invasive treatment group based on the primary treatment received. Primary outcome was symptom free survival defined as any symptoms of the affected arm (e.g., swelling, edema or pain of the upper limb, fatigue in rest or during exercise, paresthesia etc.) reported at last follow-up regardless of severity, frequency, or extent of disability caused by these symptoms. Secondary outcome measures were incidence of bleeding complications scored with the expanded WHO-bleeding classification, recurrent VTE of the affected arm, surgical complications (excluding bleeding complications), and required reinterventions (17, 18). Reinterventions were defined as unplanned, additional interventions that were performed during follow-up to treat (recurrent) symptoms or thrombosis.

Statistical analysis was performed using Statistical Package for the Social Sciences (SPSS; version 25 IBM, Armonk, NY, U.S.A.). Data with a continuous outcome were checked for a normal distribution and analyzed with the unpaired T-test or otherwise with the Mann-Whitney U test in case of a skewed distribution. Data with a categorical outcome were analyzed with the chi-square test.

## Results

A total of 115 patients with pUEDVT were included and divided into three distinct treatment strategies; A conservative treatment group (group 1, *N* = 35), an invasive treatment group (Group 2, *N* = 53) and a cross-over group of patients treated surgically after a first period of conservative therapy (Group 3, *N* = 27). The mean ages among all three groups were between 30 and 40 years old. The side of the affected arm and the percentage of patients with effort induced thrombosis (Paget-Schroetter syndrome) were similar in all groups. The median follow-up was between 36, 26, 22 months for groups 1–3 respectively (Table 1).

**Table 1:** Baseline data.

	Group 1 (*N* = 45)	Group 2 (*N* = 53)	Group 3 (*N* = 27)
Mean age (years; sd)	38.9 (13.8)	32.8 (11.7)	36.3 (13.2)
Male sex	23 (51.1%)	31 (58.5%)	13 (48.1%)
Affected side: right arm	20 (44.4%)	28 (52.8%)	16 (59.3%)
Median follow-up (mos; IQR)	36 (56)	26 (41)	22 (51)
Etiology of pUEDVT			
1. vTOS	6 (13.3%)	51 (96.2%)	27 (100%)
2. Idiopathic	4 (8.9%)	-	-
3. Missing	35 (77.8%)	2 (3.8%)	-
Effort induced thrombosis (PSS)	15 (33.3%)	17 (32.1%)	10 (37.0%)
Primary treatment:			
1. Physical therapy	1	-	-
2. Antithrombotic therapy			
• Vit K	18	-	21
• DOAC	25	-	6
• Antiplatelet	1	-	-
4. CDT + TOD	-	31	-
5. Anticoagulation + TOD	-	14	-
6. TOD	-	5	-
7. CDT	-	1	-
8. Intravenous stenting	-	2	-
Median duration of antithrombotic therapy (mos; IQR)	6 (23.5)	-	13 (27.0)^a^
Secondary treatment	NA	NA	
1. TOD	-	-	13
2. TOD + PTA	-	-	7
3. CDT + TOD	-	-	1
4. CDT + TOD + PTA	-	-	6
Postoperative antithrombotic therapy	NA		
• Vit K	-	19	8
• DOAC	-	22	8
• Antiplatelet	-	2	-
• LMWH	-	1	2
• None	-	9	9
Median duration of postoperative antithrombotic therapy (mos; IQR)	NA	6 (10.5)	2.0 (5.0)

**Legend.** CDT, catheter directed thrombolysis; DOAC, Direct Oral Anticoagulant; NA, not applicable; PSS, Paget Schroetter syndrome; TOD, thoracic outlet decompression; Vit K, Vitamin K antagonist.

^a^ Median duration of antithrombotic therapy prior to surgical intervention.

### Conservative treatment group 1

Ten patients (22.2%) received additional diagnostic imaging (CTv, MRv or angiography) besides duplex scanning to determine the etiology of the thrombosis. Six patients (13.3%) were diagnosed with vTOS, four (8.9%) with idiopathic thrombosis. In the remaining 35 patients (77.8%) no additional imaging of the thoracic outlet was performed and thus no distinction in pUEDVT etiology could be made. However, we ensured that no factors for sUEDVT such as indwelling vascular devices, malignancies or limb immobilization were present in these patients. Forty-four patients (97.8%) were treated with antithrombotics; 25 DOAC, 18 vitamin K antagonist, one antiplatelet therapy. One patient only received physical therapy focused on posture improvement without additional medicinal therapy (Table 1). There was a wide variation in duration of antithrombotic therapy ranging from six weeks to 94 months with a median length of six months (IQR 23.5 months). Sixteen patients (35.6%) were symptom free at last follow-up (median 36 months), the remaining 29 patients had persisting or recurrent symptoms (*N* = 22) or recurrent thrombosis (*N* = 7). Ten of these patients were eventually treated surgically and were included as cross-over patients of group 3 for the postoperative follow-up. Three patients suffered bleeding complications under DOAC usage; one patient had frequent but mild epistaxis (Grade 1), one had severe menorrhagia (Grade 2) and ceased anticoagulation therapy, the third patient required a blood transfusion due to severe vaginal blood loss (Grade 3) but continued anticoagulation therapy afterwards. The only patient who required a reintervention was the patient treated with physical therapy. Because of persisting symptoms a TOD and PTA was performed leading to full symptom resolution.

### Invasive therapy group 2

In group 2, five treatment categories could be distinguished. The majority of patients were treated with surgery; 31 patients (58%) with CDT followed by TOD, 14 patients (26%) with anticoagulant therapy followed by staged TOD, and five patients (9%) with TOD only. Three patients did not primarily undergo surgery but received intravenous stent placement (*N* = 2; 4%) or CDT only (*N* = 1; 2%). Additionally, 44 patients (83%) received some form of post procedural anticoagulation therapy for a median duration of six months (IQR 10.5 months); 22 patients with DOACs, 19 with vitamin K antagonists, one with low-molecular weight heparin, and two with antiplatelet therapy.

After primary treatment, 24 patients were symptom free and 29 patients had recurrent or persisting symptoms. Seven patients suffered an ipsilateral recurrent thrombosis, the remaining 22 patients were symptomatic without recurrent thrombosis (Table 2). In 14 of these symptomatic patients a total of 23 reinterventions were performed leading to symptom resolution in an additional five patients (Table 3). The majority of reinterventions were PTA's for symptomatic recurrent stenosis (*N* = 10, 12 PTA's). Three patients underwent a redo TOD; two because of an insufficiently removed first rib and one because of an accidental second rib removal during primary intervention. The patient primarily treated with CDT was relieved of symptoms after TOD. The two primarily stented patients both suffered a stent fracture and were treated with TOD and PTA leading to full symptom resolution. Finally, in three patients with PTA resistant stenosis stents were placed, leading to full symptom resolution in two. The third patient was additionally treated with a pectoralis minor release and finally a subclavian vein bypass, but remained symptomatic. Two patients (4%) suffered postoperative bleeding complications (Grade 2 & 3). Both were surgical explored without finding a clear bleeding focus. Six additional surgery related complications occurred in five patients, none led to long-term morbidity (Table 4).

**Table 2:** Symptom free survival, bleeding complications and recurrent VTE.

	Group 1 (*N* = 45)	Group 2 (*N* = 53)	Group 3 (*N* = 27)
Symptom free at last follow-up (%)	16 (35.6%)	29 (54.7%)	13 (48.1%)
Bleeding complications^a^ (%)			
1. Grade 1	1	-	-
2. Grade 2	1	1	4
3. Grade 3	1	1	1
4. Grade 4	-	-	-
Total:	3 (8.6%)	2 (3.8%)	5 (18.5%)
Patients with recurrent VTE (%)	7 (15.6%)	7 (13.2%)	4 (14.8%)
Total amount of recurrent VTE's	7	11	4

**Legend.** VTE, venous thromboembolism.

^a^ According to the expanded WHO bleeding scale.

**Table 3:** Re-interventions.

	Group 1	Group 2	Group 3
	(*N* = 45)	(*N* = 53)	(*N* = 27)
Patients with a re-intervention (%)	1 (2.2%)	14 (26.4%)	6 (22.2%)
Re-interventions specified			
1. PTA	-	12	8
2. CDT + TOD	-	1	-
3. TOD	-	-	-
4. TOD + PTA	1	2	-
5. Stenting	-	3	-
6. Redo TOD	-	3	1
7. Pectoralis minor release	-	1	-
8. Subclavian vein bypass	-	1	-
Total	1	23	9
Number of symptom free patients after re-intervention	1 (100%)	5 (35.7%)	2 (33.3%)

**Legend.** CDT, catheter directed thrombolysis; PTA, percutaneous transluminal angioplasty; TOD, thoracic outlet decompression.

Note 1: for group 3 only the re-interventions after TOD are presented here.

Note 2: For group 1, the 10 cross-over patients included into group 3 are not mentioned as reinterventions, but instead are counted as symptomatic patients at last follow-up.

**Table 4:** Perioperative complications.

	Group 2	Group 3
	(*N* = 53)	(*N* = 27)
Perioperative complications:		
1. Pneumothorax	1	-
2. Iatrogenic vascular injury	1	-
3. Seroma	1	-
4. SSI	1	-
5. Temporary neuropraxia	2	-
6. Pneumonia	-	1
Total:	6	1
Number of patients affected (%)	5 (9.4%)	1 (3.7%)

**Legend**. SSI, surgical site infection.

Note: all bleeding complications in group 2 and 3 were surgery related but are mentioned separately in Table 2.

### Cross-over group 3

Group 3 contained 10 cross-over patients previously mentioned under group 1 and 17 patients with recurrent symptoms who were referred to our hospitals for further analysis of VTOS. All 27 patients were originally treated conservatively with anticoagulants but experienced either persisting or recurrent symptoms. Thirteen patients suffered a recurrent thrombosis, (five while under anticoagulation therapy) and 14 patients had persisting or recurrent symptoms without thrombosis. Twenty-one patients were treated with vitamin K antagonists and six with a DOAC, for a median duration of 13 months prior to surgical intervention. All patients underwent dedicated diagnostic imaging (CTv, MRv or angiography) to confirm VTOS, after which TOD was performed. Eighteen patients received postoperative anticoagulation therapy for a median duration of 2 months (Table 1). Eleven patients remained symptom free after primary surgical intervention, but 16 patients had persisting symptoms (*N* = 12) or suffered recurrent thrombosis (*N* = 4) (Table 2). Six of these patients underwent additional reinterventions leading to full symptom resolution in two patients, and 13 symptom free patients in total (48.1%) at last follow-up. Bleeding complications occurred in five patients; One patient had a bleeding complication after CDT (Grade 2) and four patients suffered a haematothorax (three grade 2, one Grade 3) of which three required surgical exploration and one was treated with a chest drain. Finally, one patient developed a pneumonia that required antibiotic treatment (Table 4).

## Discussion

In this multicenter retrospective observational cohort the conservative and direct invasive treatments for pUEDVT were deemed safe with low percentage of bleeding complications. However, the conservative treatment resulted in a symptom free survival of 36% after a median follow-up of 36 months. In the group of direct invasive treatment just over half of the patients had a symptom free survival after a median follow-up of 26 months. In the invasive treatment a total of 23 reinterventions were required in one quarter of patients. Surgery related complications occurred in less than 10 percent of patients, seeming mostly modest and did not lead to long term morbidity.

This analysis confirms that conservatively treated patients with recurrent symptoms and proven VTOS can benefit from TOD, as was shown in other studies (9). The delayed surgery group had a symptom free survival of 48% after approximately two years. Hence, it is considered worthwhile to perform additional diagnostic imaging to look for VTOS in conservatively treated symptomatic pUEDVT patients with persisting functional limitations and physical complaints. Noteworthy is that surgical bleeding complications seem to occur more frequently in this group. Theoretically one could argue that this might be caused by increased, severe scarring of the subclavian vein and surrounding tissue due to chronic compression, leading to an increasingly difficult surgical procedure.

Recommendations on the therapy of choice for pUEDVT and the use of thrombolysis or TOD vary between current guidelines, mainly due to a lack of randomized trials comparing both strategies. The CHEST, ISTH and ESC guidelines advocate anticoagulation therapy for at least three to six months and to only consider thrombolysis and TOD in selected cases with severe limb threatening thrombosis and evident symptoms of VTOS (19–21). Whereas the recent ESVS guideline is more prone towards the use of thrombolysis and TOD when clear evidence for VTOS is present (22). This contradiction is reflected by the variety of treatment strategies applied in this case series. This study also shows a significant variety in anticoagulant usage in all three groups. Naturally, the retirement of the vitamin K antagonist can easily be explained by the introduction of the DOACs in the past decade. However, we found a wide range in therapy duration and a considerable percentage of patients were put on prolonged or indefinite anticoagulation therapy contrary to the 3–6 months advocated by the majority of guidelines. As for the invasive treatment groups, the ideal duration of postoperative anticoagulant usage or even the necessity of postoperative anticoagulation therapy remains unclear. This resulted in a wide variation of postoperative antithrombotic treatment strategies based on the surgeons preference. Also worth addressing is the use of stents in this case series. A total of five patients with VTOS received primary stent placement (*N* = 2) or postoperative stent placement (*N* = 3) with mixed results. This was in line with our recently published case series and literature review, where we found that both venous and arterial stents in the thoracic outlet have a considerable risk of failure, especially in patients who were stented without prior TOD (4). Moreover, due to a limited amount of evidence and reported mixed results, stent placement in the thoracic outlet is discouraged in the most recent ESVS guideline (22).

A limitation not just of our study, but every study on pUEDVT so far, and more importantly for daily practice, is the lack of an unambiguous and reproducible scoring method for PTS in the upper extremity. Although modifications of the Villalta score, the Derkash classification, and functional disability scores such as the DASH score have been used, these are not validated to detect the presence and severity of PTS in the upper extremity. We therefore choose to report symptoms at last follow-up based on whether patients still experienced any form of residual symptoms, even if symptoms greatly improved after treatment. We believe this strict definition explains the higher percentages of symptomatic patients found across all groups compared to other large series (9–14). The percentage of patients with bleeding complications or surgery related complications on the other hand were relatively low in this study.

Strikingly, almost 80 percent of patients in the conservative group did not receive diagnostic imaging on top of duplex scanning to determine the etiology of the pUEVDT. Thus we were unable to directly compare the conservative with the invasive treatments for VTOS since the etiology of the thrombosis was unclear for the majority of conservatively treated patients. We believe this is partly caused by the fact that the guidelines make no distinction in the duration of anticoagulant treatment for any form of pUEDVT and thus additional diagnostic imaging has no treatment consequences for the conservative treatment. However, since the invasive treatment has become an increasingly common and successful approach for the treatment of VTOS and in order to provide the patient with a clear choice between treatment options, we believe additional diagnostic imaging of the thoracic outlet should be considered more often in pUEDVT patients.

### Future perspectives

Most patients with pUEDVT are young, between 25 and 35 years old, and active participants in society. PTS can severely impact their QoL and their ability to participate in society with an unequivocal socio-economic impact (5). Yet, it still remains unclear how we should treat these patients to prevent PTS. Hence, future research in the form of randomized controlled trials or prospective registries that compare the conservative and invasive treatment of pUEDVT with clearly defined outcome measures such as the recently developed UE-PTS score, are highly warranted (23, 24).

## Conclusion

In this multicenter retrospective observational cohort analysis the conservative and direct invasive treatments for pUEDVT were deemed safe with low percentages of bleeding complications. Symptom free survival was highest in the direct surgical treatment group but still modest in all subgroups. Perioperative complications were infrequent with no related long term morbidity. Of relevance, pUEDVT patients with confirmed VTOS and recurrent symptoms after conservative treatment may still benefit from delayed TOD surgery. However, the symptom free survival of this delayed surgical treatment seems lower than direct surgical treatment and bleeding complications occur more frequently.

## Data Availability

The raw data supporting the conclusions of this article will be made available by the authors, without undue reservation.
